# SG-APSIC1130: Zero wet pack Pimporn Sirikraiwattanawong, Thailand

**DOI:** 10.1017/ash.2023.98

**Published:** 2023-03-16

## Abstract

**Objectives:** We noted moisture in Thompson retractor sets after steam sterilization in our hospital. Moisture can cause severe problems leading to potentially contaminated instruments that carry infection risk to patients and cause procedure delays, wasted time and effort, greater workload, and higher costs. We sought to reduce the number of retractor sets with moisture to zero. **Methods:** The central sterile supply (CSS) team discussed the cause of the problem. We hypothesized that temperature difference between the sterilizer chamber and inside the container might create condensation and thus moisture in the final surgical set. We collected and analyzed data and proposed an experiment to improve the sterilization process. We performed a trial of sterilization process improvements pertaining to proper loading technique and the packaging process. We also evaluated the appropriate drying time for rigid containers. We then rearranged the process and adjusted the cooling time from 30 to 60 minutes after steaming. **Results:** Moisture in Thompson retractor packs occurred because of thicker, rigid containers. We removed the previous type of lining material to separately steam the rigid surgical instrument, and we extended the cooling time to 60 minutes. We updated standard operation procedures and continued to monitor and re-evaluate the process. **Conclusions:** We identified the primary cause of moisture in Thompson retractor sets after steam sterilization. We illustrated that avoiding sterilizer overload, avoiding contact with fabric wrapping materials, and proper cooling time kept the pack moisture free. Occurrences of moisture in surgical packs after sterilization should be reported and handled efficiently by CSS personnel to preserve quality and avoid waste.

